# Associations between an Obesity Related Genetic Variant (*FTO* rs9939609) and Prostate Cancer Risk

**DOI:** 10.1371/journal.pone.0013485

**Published:** 2010-10-19

**Authors:** Sarah J. Lewis, Ali Murad, Lina Chen, George Davey Smith, Jenny Donovan, Tom Palmer, Freddie Hamdy, David Neal, J. Athene Lane, Michael Davis, Angela Cox, Richard M. Martin

**Affiliations:** 1 School of Social and Community Medicine, University of Bristol, Bristol, United Kingdom; 2 MRC Centre for Causal Analysis in Translational Epidemiology, School of Social and Community Medicine, University of Bristol, Bristol, United Kingdom; 3 Nuffield Department of Surgery, John Radcliffe Hospital, Oxford, United Kingdom; 4 Department of Oncology, University of Cambridge, Addenbrooke's Hospital, Cambridge, United Kingdom; 5 Institute for Cancer Studies, School of Medicine and Biomedical Sciences, University of Sheffield, Sheffield, United Kingdom; Peninsula Medical School, United Kingdom

## Abstract

Observational studies suggest that obese men have a lower risk of incident prostate cancer, but an increased risk of advanced and fatal cancers. These observations could be due to confounding, detection bias, or a biological effect of obesity. Genetic studies are less susceptible to confounding than observational epidemiology and can suggest how associations between phenotypes (such as obesity) and diseases arise. To determine whether the associations between obesity and prostate cancer are causal, we conducted a genetic association study of the relationship between a single nucleotide polymorphism known to be associated with obesity (*FTO* rs9939609) and prostate cancer. Data are from a population-based sample of 1550 screen-detected prostate cancers, 1815 age- and general practice matched controls with unrestricted prostate specific antigen (PSA) values and 1175 low-PSA controls (PSA <0.5 ng/ml). The rs9939609 A allele, which was associated with higher BMI in the sample, was inversely associated with overall (odds ratio (OR) versus all controls  = 0.93; 95% confidence interval (CI): 0.85–1.02 p = 0.12 per allele) and low-grade (OR = 0.90; 0.81–0.99 p = 0.03 per allele) prostate cancer risk, but positively associated with high-grade cancer among cases (OR high- versus low-grade cancer  = 1.16; 0.99–1.37 p = 0.07 per allele). Although evidence for these effects was weak, they are consistent with observational data based on BMI phenotypes and suggest that the observed association between obesity and prostate cancer is not due to confounding. Further research should confirm these findings, extend them to other BMI-related genetic variants and determine whether they are due to detection bias or obesity-related hormonal changes.

**Trial Registration:**

Controlled-Trials.com ISRCTN20141297

## Introduction

Prostate cancer is a major cause of morbidity and mortality worldwide [1]. Advancing age, skin colour and a family history of prostate cancer are known predisposing factors [2], but little is known about modifiable risk factors for the disease. Knowledge of such factors may aid in the development of preventative and treatment strategies. Since obesity has been found to be a risk factor for many forms of cancer [3], and since it is highly prevalent among westernized societies, it seems reasonable to investigate whether it could also be a risk factor for prostate cancer.

Observational studies of obesity and prostate cancer have produced mixed results. A meta-analysis published in 2006 of 22 prospective cohort studies found that obesity was associated with a small increase in prostate cancer risk [4]. However, when a stratified analysis was carried-out, the authors found that the increase was limited to advanced rather than localised disease [4]. Since this meta-analysis, several studies have been published which indicate that obesity is associated with an increased risk of advanced or fatal prostate cancer, but with a decreased risk of localised disease [5]–[9].

There are several potential explanations for these findings. They may have arisen as a result of confounding by factors such as diabetes mellitus. Obesity strongly predisposes to type 2 diabetes mellitus (T2DM) and epidemiological studies (including our own [10]) have consistently reported an inverse association between T2DM and prostate cancer (meta-analysis pooled relative risk, RR = 0.84, 95% CI: 0.76–0.93) [11].

A further explanation could be that obesity makes prostate cancer identification more difficult, thus predisposing obese men to present later with more severe disease, but reducing the identification of low-grade, low volume disease (detection bias, generating positive association with advanced cancer but inverse associations with localised disease). This possibility has been suggested because digital rectal examination is technically more difficult in obese patients [9], biopsies are more likely to lead to false negative findings due to enlarged tissue [9] and epidemiological studies have found that prostate specific antigen (PSA) concentrations are lower in obese than in non-obese men [12]–[14], possibly due to greater plasma volume in obesity resulting in haemodilution and therefore lower relative concentrations of PSA [15]. However, the recent Prostate Cancer Prevention Trial reported that differences in cancer grade and stage were maintained even amongst a cohort of men who all underwent prostate biopsy [5], suggesting that detection bias due to lower PSA levels among obese individuals cannot account for all of the observed effects. In addition, obesity was positively associated with clinical progression in men with prostate cancer (i.e. who all underwent prostate biopsy), independent of cancer grade, stage and primary treatment [16].

Whilst detection of prostate cancer may be more difficult amongst obese men, the treatment received by those obese patients who *do* develop prostate cancer may be less effective than that received by non-obese patients, for example, as a result of technical difficulties during surgery [17] or difficulty in targeting radiotherapy [18].

It is also possible that hormonal changes associated with obesity increase the proliferative potential of prostate cancer. *In vitro* and epidemiological studies have demonstrated that steroid hormones, leptin and insulin-like growth factor-1 (IGF-1), all of which are raised among obese individuals, increase prostatic tumour cell proliferation [19], [20].

In summary, whilst epidemiological studies have identified differences in prostate cancer risk between obese and non-obese men, the possibility of confounding and bias means that a causal effect of obesity on prostate cancer risk has not yet been conclusively demonstrated. Genetic studies are less susceptible to confounding than observational epidemiology [21] and may offer a complimentary study design [22]. The existence of genetic variations that alter risk of developing both obesity *and* prostate cancer could constitute evidence in favour of a causal link between the two diseases.

A single nucleotide polymorphism (SNP), known to be associated with obesity (*FTO* rs9939609), has been robustly associated with increased body mass index (BMI) and obesity in multiple study populations [23]–[26]. It has been suggested that this effect is mediated through a reduction in satiety [27] and consequent increased food consumption [28]. The rs9939609 SNP may, therefore, present an un-confounded exposure with which to investigate the causal association between obesity and prostate cancer.

We hypothesised that the AA genotype of rs9939609, which is associated with an increase in BMI, would protect against non-aggressive prostate tumours whilst increasing the risk of aggressive prostate tumours. To test this hypothesis, we present data from a large, case-control study nested within the population-based phase of the ProtecT (Prostate testing for cancer and Treatment study) trial.

## Materials and Methods

### Study participants

Participants in this study were selected from the Prostate testing for cancer and Treatment study (ProtecT), which is a randomized controlled trial taking place in nine regions of the UK with the aim of evaluating the efficacy, cost effectiveness, and acceptability of treatments for localized prostate cancer. All men aged 50–69 years from approximately 300 general practices and without known prostate cancer, were invited to attend a nurse-led prostate check clinic and have a PSA test. The invitations were sent between 2001 and 2008 and over 89,000 men attended the prostate check clinic. Participants with a single raised PSA level over 3.0 ng/ml were invited to attend the centre's urology department for digital rectal examination (DRE), repeat PSA test, and transrectal ultrasound (TRUS) guided biopsy (10 cores), or referred to a urologist if the PSA level was over 20 ng/ml, to confirm prostate cancer status. The age of the participants when they attended the prostate check clinic, PSA level, height, weight, smoking status, physical exercise and self-reported ethnicity and diabetes were collected either by questionnaire or by nurse interview. In this study, our case population consisted of all men with prostate cancer identified at prostate check clinics conducted before the end of November 2006 who gave consent for genotyping.

Prostate cancer stage was defined according to the 2002 TNM staging system [29]. Patients with cancer stage between T0-T2 were defined as having localized stage cancer, while individuals with a cancer stage greater than T2 were defined as advanced stage cases. Histological cancer grade was defined by Gleason score using grade 7 as the cut-off (lower grade: <7; higher grade: ≥7). We found little overlap between more aggressive prostate cancer cases defined by these two methods. Only 19.4% of patients with Gleason score ≥7 have cancer stage ≥T2, whereas 72.2% of patients at stage ≥T2 have Gleason score ≥7.

Two non-overlapping groups of controls were randomly selected from the pool of men who attended the prostate check clinics and did not have prostate cancer diagnosed. One control group included only participants without a diagnosis of prostate cancer and with a PSA concentration <0.5 ng/ml (‘low PSA/super-normal controls’). The other group included only participants without a diagnosis of prostate cancer and placed no restriction on PSA concentration (‘unrestricted controls’). Unrestricted controls were stratum matched to cases by age (5-year bands) and the primary care centre (general practice) from which men were recruited; low PSA controls were matched to cases where possible, but if a matched low PSA control was not available, an unmatched low PSA control was selected. Multi-centre Research Ethics Committee approval was obtained from Trent MREC, and written consent for performing anonymized genotyping on stored blood was obtained from individual participants. Detailed descriptions of the ProtecT study and the protocol for nested case-control selection are published elsewhere [30]–[32].

### DNA extraction and genotyping

DNA extraction was performed by Tepnel (http://www.tepnel.com). The *FTO* rs9939609 variant was genotyped in ProtecT participants as part of a genetic association study examining the effect of 70 diet/nutrition relevant SNPs on prostate cancer risk and was undertaken by KBioscience Ltd (www.kbioscience.co.uk), who use their own form of competitive allele-specific PCR (KASPar) and Taqman™, for SNP analysis. Samples with more than 10% genotype failure (7 SNPs) were defined as having poor DNA quality (2.6%) and dropped from further analysis. Genotyping was repeated in 10% of the study samples (with independent assessment) and for 99.98% of those samples there was exact agreement between the two.

### Statistical analysis

A Pearson χ^2^-test was performed amongst controls to ensure that genotype distribution satisfied Hardy-Weinberg equilibrium. We tested for differences in demographic and lifestyle characteristics between cases and controls and between low PSA and other controls. We also tested for differences in these characteristics between genotypes. We used Student's t-test for quantitative variables, such as age, BMI, PSA level (log transformed), exercise intensity scores and weekly drinking and χ^2^ tests for ordered categorical variables, such as smoking status (current smoker, ever smoker and non-smoker) and social class (professional, intermediate and manual). Associations of rs9939609 with all prostate cancers and prostate cancer stage or grade were calculated using unconditional logistic regression models adjusted for exact age at prostate check clinic and study centre (9-level variable).

Instrumental variable (IV) estimation of the effect of BMI on prostate cancer was performed by dividing the genotype-outcome log odds ratios by the genotype-BMI association from the controls, known as the Wald type estimator or ratio of coefficients approach [33], [34]. This estimate was exponentiated to give a causal odds ratio per unit change in BMI. The standard error of the IV estimate on the log scale was calculated using a Taylor series of the ratio of two means [35] Statistical analyses were carried out in Statastatistical software (version 10; Stata Corporation, College Station, TX). P-values are two-sided.

## Results

DNA samples from 4664 participants were submitted for genotyping. We excluded 91 individuals who reported being of ethnicities other than white European in an attempt to avoid population stratification in our analysis. The distributions of co-variables in the remaining population are outlined in **Table 1**. Cases were more likely to have a family history of cancer than controls and more likely to be non-smokers, but did not differ in relation to BMI, waist-hip ratio, exercise intensity score, alcohol intake, or social class. Low PSA controls were younger, had a higher BMI and drank more alcohol than the ‘unrestricted’ controls.

**Table 1:** Characteristics of the study population.

	Matched ‘unrestricted’ controls	Prostate cancer cases	*p-*value	Low PSA controls	*p*-value
No. of participants	1824	1566	—	1183	—
Family history					
Yes	95 (5.2)	116 (7.4)	0.008	46 (3.9)	0.10
No	1730 (94.8)	1450 (92.6)		1137 (96.1)	
Age in years (mean±sd), year	62.7±5.0	62.5±5.1	0.36	60.8±5.4	<0.0001
PSA (mean±sd)*, ng/ml	1.3±1.4	9.3±26.1	<0.0001	0.36±0.1	N/A
BMI (mean±sd), kg/m^2^	26.8±3.7	26.7±3.6	0.41	27.5±4.1	0.0001
WHR (mean±sd), unit	0.93±0.002	0.93±0.002	0.95	0.93±0.002	0.99
Exercise intensity scores (mean±sd)*, unit	22.6±33.6	23.2±34.9	0.07	22.8±33.6	0.75
Weekly drinking (mean±sd)*, unit	19.0±17.8	17.7±16.7	0.09	21.7±19.8	0.009
Social class (n,%)					
Professional	722 (44.0)	721 (46.7)	0.26	398 (47.9)	0.18
Intermediate	279 (15.9)	245 (15.8)		122 (14.7)	
Manual	703 (40.1)	579 (37.5)		311 (37.4)	
Smoking (n, %)					
Non-smoker	405 (32.6)	430 (37.3)	0.045	277 (32.7)	0.75
Ever smoker	661 (53.3)	561 (48.6)		441 (52.1)	
Current Smoker	175 (14.1)	163 (14.1)		129 (15.2)	

‘Unrestricted’ controls are the baseline group for comparison,

*p-values are calculated based on log transformed data.

Genotyping was successfully carried out for 1550 of 1566 (99.0%) cases, 1815 out of 1824 (99.5%) controls, and 1175 of 1183 (99.3%) controls. Genotypes conformed to Hardy-Weinberg equilibrium in all 3 groups (cases p = 0.91, ‘unrestricted’ controls p = 0.36, low PSA controls p = 0.14). Four hundred and forty nine out of 1545 men in whom histological grade was confirmed were defined as having high-grade cancer, whereas only 196 out of 1546 cases were defined as having advanced-stage cancer.

**Table 2** shows the association between the *FTO* rs9939606 genotype and the baseline characteristics of the ‘unrestricted’ control population. There were no differences in the distribution of these variables by genotype; they cannot, therefore, confound the association between genotype and disease risk.

**Table 2:** Characteristics of the matched ‘unrestricted’ control population by *FTO* rs9939609 genotype.

	TT	TA	*p-*value	AA	*p*-value
Total No. of participants	676	848	—	291	—
Family history					
Yes	39 (5.8)	42 (5.0)	0.48	14 (4.8)	0.55
No	637 (94.2)	806 (95.1)		227 (95.2)	
Age in years (mean±sd), year	63.0±5.1	62.4±5.0	0.03	62.9±5.0	0.69
PSA (mean±sd)*, ng/ml	1.3±1.3	1.3±1.5	0.89	1.2±1.3	0.30
BMI (mean±sd), kg/m^2^	26.5±3.6	27.0±3.7	0.07	26.9±4.0	0.22
WHR (mean±sd), unit	0.93±0.06	0.94±0.06	0.17	0.94±0.06	0.31
Exercise intensity scores (mean±sd)*, unit	23.5±33.8	20.5±28.8	0.43	26.7±40.1	0.21
Weekly drinking (mean±sd)*, unit	18.5±16.7	20.5±19.3	0.23	15.4±14.4	0.36
Social class (n,%)					
Professional	276 (42.8)	369 (45.2)	0.65	123 (43.5)	0.97
Intermediate	106 (16.4)	127 (15.6)		45 (15.9)	
Manual	263 (40.8)	320 (39.2)		115 (40.6)	
Smoking (n, %)					
Non-smoker	159 (32.9)	179 (32.8)	0.71	65 (31.7)	0.80
Ever smoker	252 (52.0)	294 (53.9)		112 (54.6)	
Current Smoker	73 (15.1)	73 (13.3)		28 (13.7)	

The TT genotype is the baseline category for comparison,

*p-values are calculated based on log transformed data.

**Table 3** shows the association between genotype and mean BMI among cases, ‘unrestricted’ controls and ‘low-PSA’ controls. The difference between AA and TT genotypes was similar among the 3 groups with an overall difference of 0.56 kg/m^2^ (p = 0.007). We also assessed the association between genotype and log PSA level among the cases and normal (‘unrestricted’) controls (**Table 4**), but not amongst the low PSA controls (because these were selected to have extremely low PSA levels (<0.5 ng/ml) which is around the limit of detection and it is therefore likely that the ability to detect differences in PSA levels by genotype will be low in this group). We found no strong statistical evidence of any differences in log PSA level by genotype (mean log PSA differences comparing AA versus TT were −0.07 and −0.06 amongst cases and controls, respectively).

**Table 3:** Association between BMI and *FTO* genotype (adjusted by age and centre).

	TT	TA	Mean difference, 95% CI (TA minus TT)	*p*	AA	Mean difference, 95% CI (AA minus TT)	*p*	per allele effect	*p*
**Low PSA Controls**	27.3±4.0	27.4±3.9	0.05 (−0.57, 0.68)	0.86	28.1±4.7	0.77 (−0.06, 1.61)	0.07	0.32 (−0.08, 0.73)	0.12
**Matched ‘unrestricted’ controls**	26.5±3.6	27.0±3.7	0.44 (−0.04, 0.91)	0.07	26.9±4.0	0.39 (−0.24, 1.03)	0.22	0.24 (−0.06, 0.55)	0.11
**Cancer Cases**	26.5±3.5	26.6±3.6	0.08 (−0.39, 0.55)	0.74	27.1±3.6	0.49 (−0.16, 1.15)	0.14	0.21(−0.10, 0.52)	0.19
**All controls**	26.8±3.8	27.1±3.8	0.29 (−0.9, 0.67)	0.13	27.4±4.3	0.57 (0.06, 1.07)	0.03	0.28 (0.04, 0.53)	0.02
**Low grade cancers**	26.3±3.2	26.7±3.7	0.39 (−0.16,0.94)	0.16	27.4±3.6	1.04 (0.25, 1.83)	0.01	0.49 (0.12, 0.86)	0.01

CI  =  confidence interval.

**Table 4:** Association between log PSA and *FTO* genotype.

	TT	TA	Mean difference	*p*	AA	Mean difference	*p*	per allele effect	*p*
**Matched Controls**	−0.09±0.85	−0.10±0.83	−0.006 (−0.09, 0.08)	0.89	−0.15±0.85	−0.06 (−0.18, 0.05)	0.30	−0.03 (−0.08, 0.03)	0.36
**Cancer Cases**	1.85±0.68	1.81±0.72	−0.03 (−0.11,0.05)	0.43	1.78±0.62	−0.07 (−0.17, 0.03)	0.19	−0.03 (−0.08, 0.02)	0.20

**Table 5** shows the results of our analyses of associations between genotype and prostate cancer risk. Those with the A allele had a lower odds of all- or low-grade cancers, compared to those with the TT genotype (p-values for per allele effects were between 0.03 and 0.18). The results for all cases versus all controls (‘unrestricted’ plus ‘low-PSA’) suggested a 7% reduction per A allele (95% CI = −2% to 15%). There was evidence of a 10% reduction (1% to 19%) in risk of low grade prostate cancer per A allele (p = 0.03). There was no association with having high grade or advanced stage cancer per se, but among cases the results for high-grade versus low-grade cancer suggested that risk of high grade disease was increased by 16% per A allele (95% CI-1% to 37%, p = 0.07). However, p-values were not sufficiently small to provide strong evidence against the null hypothesis. Adjustment by whether men had diabetes or not (self-report) made no difference to the results (not shown).

**Table 5:** Association between *FTO* genotype and prostate cancer outcomes adjusted by age and study centre.

	TA vs TT		AA vs TT		per allele effect	
	Odds Ratio (95%CI)	*p*	Odds Ratio (95%CI)	*p*	Odds Ratio (95%CI)	*p*
**Cancer cases (n = 1550) vs matched controls (n = 1815)**	0.96 (0.83–1.12)	0.62	0.86 (0.70–1.06)	0.16	0.94 (0.85–1.03)	0.18
**Cancer cases (n = 1550) vs low PSA controls (n = 1175)**	0.99 (0.83–1.17)	0.87	0.82 (0.65–1.03)	0.09	0.92 (0.82–1.03)	0.14
**Cancer cases (n = 1550) vs all controls (n = 2990)**	0.98 (0.85–1.12)	0.73	0.84 (0.70–1.02)	0.08	0.93 (0.85–1.02)	0.12
**Low grade cancers (n = 1096) vs all controls (n = 2990)**	0.94 (0.80–1.09)	0.38	0.78 (0.63–0.97)	0.02	0.90 (0.81–0.99)	0.03
**Localized stage (n = 1350) vs all controls (n = 2990)**	0.95 (0.83–1.10)	0.50	0.87 (0.71–1.05)	0.15	0.94 (0.85–1.03)	0.16
**High grade (n = 449) vs low grade (n = 1096)**	1.20 (0.94–1.54)	0.14	1.33 (0.95–1.88)	0.10	1.16 (0.99–1.37)	0.07
**Advanced stage (n = 196) vs localized stage (n = 1350)**	1.27 (0.91–1.77)	0.16	0.79 (0.47–1.34)	0.38	0.99 (0.79–1.23)	0.90
**High grade (n = 449) vs all controls (n = 2990)**	1.09 (0.87–1.36)	0.45	1.01 (0.75–1.37)	0.94	1.02 (0.89–1.18)	0.78
**Advanced stage (n = 196) vs all controls (n = 2990)**	1.14 (0.83–1.57)	0.40	0.68 (0.41–1.12)	0.13	0.91 (0.73–1.12)	0.36

Instrumental variable estimates (**Table 6**) gave an OR of 0.77 (95% CI 0.52, 1.15) for prostate cancer per unit increase in BMI (using the per allele estimates for genotype) and an OR of 1.35 (95% CI 0.90, 2.03) for high-grade versus low-grade cancer per unit increase in BMI.

**Table 6:** Instrumental variable (IV) estimates of the effect of BMI on prostate cancer.

Comparison group	Cases	OR, 95% CI (TA versus TT)	*p*	OR, 95% CI (AA versus TT)	*p*	OR, 95% CI (additive genotype model)	*p*
**Low PSA Controls**	Cancer cases	0.82 (0.01, 57.59)	0.93	0.60 (0.22, 1.66)	0.33	0.77 (0.47, 1.25)	0.29
**Matched ‘unrestricted’ controls**	Cancer cases	0.91 (0.64, 1.30)	0.61	0.68 (0.30, 1.55)	0.36	0.77 (0.46, 1.30)	0.33
**All controls**	Cancer cases	0.93 (0.56, 1.56)	0.79	0.74 (0.48, 1.13)	0.16	0.77 (0.52, 1.15)	0.20
**Low grade cancers**	High grade cancer	1.60 (0.64, 3.98)	0.32	1.32 (0.89, 1.94)	0.17	1.35 (0.90, 2.03)	0.14

OR  =  odds ratio, CI  =  confidence interval.

## Discussion

To our knowledge, this is the first study to look for a possible association between a SNP predisposing to obesity and prostate cancer. Our large, population-based, nested case-control study has found weak evidence that the rs9939609 A allele, which has previously been associated with obesity and is associated with raised BMI amongst our study population, protects against prostate cancer incidence, and stronger evidence that the same genotype protects against low-grade prostate cancer. This supports findings from epidemiological studies including our own [36], which have noted that obesity protects against localised prostate cancer. Our study found some evidence that rs9939609 A allele increases the risk of high-grade versus low-grade cancer among cases but FTO genotype was not associated with the presence of high grade or advanced stage disease per se (i.e. in comparison with non prostate cancer controls). In line with these findings, we did not find an association between BMI and high grade or advanced stage prostate cancer or even with prostate cancer risk overall in our observational analysis [36].

Whilst our effect sizes by genotype were relatively modest, it is important to note that the effect of genotype on BMI was also modest with a per allele difference of 0.28 kg/m^2^. The instrumental variable analysis suggests that prostate cancer risk is reduced by 23% per BMI unit, which would be quite substantial, although confidence intervals around this effect are wide reflecting both the uncertainty in the genotype-BMI association and the uncertainty in the genotype-prostate cancer association.

In our sub group of the ProtecT study (for which DNA was available) we did not find any difference in mean BMI between cases and controls, but a more comprehensive analysis not restricted to those with DNA found an inverse association of increasing BMI with localised cancer (unpublished work) suggesting that power may have been an issue in our study. Approximately one third of men included in our genotype analysis did not report their BMI and so could not be included in the BMI - cancer association analysis presented in Table 1. If there were a tendency for overweight men to not report their BMI, then this would bias our observational results, but not our genotype results (as very few eligible men were excluded from the genotype analysis) towards the null.

It is important to note that unlike other studies which found that high grade or advanced stage cancer were more prevalent among men with a higher BMI, we found BMI to be associated with prostate cancer grade among cases, but we found no difference in genotype distributions (and therefore BMI) between high grade cancers and controls. Also, although we measured more aggressive prostate cancer using both Gleason grade and TNM staging, the strongest effects in our study were found using a classification of Gleason grade. We observed similar effects in an earlier analysis of vitamin D and prostate cancer progression [we reported and discussed this in detail in ref 36]. In a study comparing the predictive ability of the two systems for classifying prostate cancer, Gleason score has been reported to have higher predictive accuracy for biochemical recurrence compared with TNM staging [37]. Interestingly, in the meta-analysis of BMI and prostate cancer by MacInnes and English [4], the study which showed the largest effect of BMI on increased risk of more aggressive cancer was that which used Gleason grading; all other studies used TNM staging and their results were all compatible with no increased risk. Alternatively the fact that we and others found no evidence of an increase risk for advanced stage among men with prostate cancer could simply be due to low power to detect an effect. In this study there were only 196 men in the advanced stage group, as opposed to 449 in the high grade group. Confidence intervals for the effect estimates for both high grade and advanced stage were overlapping and compatible with there being an increased risk for advanced stage as well as high grade cancer. We had around 57% power to detect a 10% increase or decrease in prostate cancer risk per FTO allele, but only around 14% power to detect a similar effect with advanced stage cancer. Future studies would need to be of the order of 2700 cases (or advanced cases, depending on the question) to have 80% power to detect an effect of this order with FTO genotype.

Our study has several advantages over traditional epidemiological studies. Genetic studies of disease risk are less susceptible to confounding and reverse causation and we can therefore be reasonably confident that the protective effect of the rs9939609 genotype is not due to these factors. In this study adjustment by whether men reported having diabetes or not made no difference to our results, suggesting that diabetes is not a confounder in the association between FTO genotype and prostate cancer risk. This is in line with Gong et al [5] who found in their observational study that the association between obesity and prostate cancer risk was independent of diabetes. Furthermore, whilst we cannot rule out the possibility that our results are due to detection bias, the lack of an effect of rs9939609 on PSA concentration (Table 4) suggests that PSA levels did not play a major role in our findings. If we ignore the p-values and apply a correction factor based on the mean difference in log PSA levels between controls with the AA and those with the TT genotype, we find that under-detection due to a lower PSA level among AA individuals cannot explain our results. The mean difference in log PSA levels between AA and TT genotypes was −0.06 units; this equates to a geometric mean difference of 0.94, i.e. a 6% difference on a log scale. If we were to lower our PSA cut-off by 6% among AA individuals we would have biopsied a further 3 individuals. Since the chance of detecting prostate cancer among biopsied individuals in our study was 25%, we would expect 0.75 cases to have been missed due to lower PSA levels among AA individuals. This would not affect the results of our analysis of genotype and cancer risk. In addition, because our prostate cancer cases came from a population based cohort study our results are unlikely to have arisen by survivor bias. However, whilst PSA detection bias is unlikely to explain our results, it is possible that detection bias due to biopsy could have occurred. It has been suggested that biopsies among obese men are more likely to lead to false negatives due to enlarged tissue [9]. In future studies it will be important to clarify the degree to which detection bias is responsible for associations between BMI and prostate cancer and whether there is another causal mechanism which is responsible for the observed association. These issues could be addressed by more thorough examination and a greater number of biopsies among obese men and also by investigating the extent to which other pathways such as hormonal pathways could explain the association.

Mendelian randomization offers evidence that is complimentary to that provided by conventional observational epidemiology, and can avoid confounding by lifestyle factors and bias due to reverse causation [21]. However it is important to note that there are limitations to this approach. The biological consequences of variation at the FTO locus and the mechanism of the observed association of this with fat mass are still unclear. Several studies exist which point to a role for this locus in energy regulation and hypothalamically regulated patterns of appetite [27]–[28], [38]–[42]. However, the possibility of pleiotrophy in the association between FTO genotype and prostate cancer risk cannot be completely ruled out. In this case, utilizing multiple instruments that is, several independent genetic variants that are associated with BMI could help strengthen the causal inference, as pleiotropic effects are unlikely to influence the effects of each instrument in the same manner [22]. In addition it is possible to generate multiple combinations of genetic variants that are independent of each other to generate many independent variable estimates as described in [43]. Future studies using multiple genetic instruments could also use combinations of alleles, as allele scores, to increase power and strengthen the IV estimation [44].

In conclusion, our data provides some evidence (albeit weak) that the A allele of rs9939609 may protect against prostate cancer risk or reduce the likelihood of this disease being detected (in particular low-grade cancer), but may increase the likelihood of cases having high grade as opposed to low grade prostate cancer at diagnosis. These observations support the findings from epidemiological studies that obesity protects against localised prostate cancer but increases the risk of advanced cancer. Further studies of this SNP and investigations of other obesity associated polymorphisms are required to provide clarity in this area.
